# SARS-CoV-2: Molecular Structure, Pathogenesis, Potential Therapeutic Targets, and Immune Response of the Infected Subject

**DOI:** 10.1155/2022/7856659

**Published:** 2022-06-02

**Authors:** R. Wumba, M. Mandina, Y. Nlandu, J. R. Makulo, A. Tshimpi, P. Mbala, A. Mbangama, P. Kabututu, J. M. Kayembe

**Affiliations:** ^1^Service of Parasitology, Department of Tropical Medicine, Infectious and Parasitic Diseases, University of Kinshasa, Kinshasa, Democratic Republic of the Congo; ^2^Service of Infectious and Parasitic Diseases, Department of Internal Medicine, University of Kinshasa, Kinshasa, Democratic Republic of the Congo; ^3^Service of Nephrology, Department of Internal Medicine, University of Kinshasa, Kinshasa, Democratic Republic of the Congo; ^4^Service of Gastroenterology, Department of Internal Medicine, University of Kinshasa, Kinshasa, Democratic Republic of the Congo; ^5^Service of Microbiology, Department of Medical Biology, University of Kinshasa, Kinshasa, Democratic Republic of the Congo; ^6^National Institute for Biomedical Research (INRB), Kinshasa, Democratic Republic of the Congo; ^7^Service of Obstetrical, Department of Gynecology and Obstetrical, University of Kinshasa, Kinshasa, Democratic Republic of the Congo; ^8^Service of Molecular Biology, Department of Basic Sciences, Faculty of Medicine, University of Kinshasa, Kinshasa, Democratic Republic of the Congo; ^9^Service of Pneumology, Department of Internal Medicine, University of Kinshasa, Kinshasa, Democratic Republic of the Congo

## Abstract

**Background:**

The pathogenic mechanisms and immune response of COVID-19 are far from clear. Through a documentary review of literature, the authors describe virological and molecular aspects of SARS-CoV-2, the intimate mechanisms of cell infection, and potential therapeutic targets. They also analyze the characteristics of immune response of the infected subject.

**Objectives:**

Objectives of this study are to describe the state of knowledge on virological data, molecular and physiopathogenic mechanisms of SARS-CoV-2, with a view to a better understanding of the therapeutic targets, as well as the immune response of the infected subject. *Methodology*. This documentary review is a compilation of several meta-analyses, consistent with the methodology described in the PRISMA statement on literature data on SARS-CoV-2, published between March 22 and August 14, 2020 (Moher et al.). The search engines used for the selection of articles were as follows: PubMed, Google Scholar, Global Health, and WHO reports. Papers of interest were those addressing virological and molecular data on SARS-CoV-2, therapeutic aspects of COVID-19, and immunity of the infected subject. Of the 617 eligible papers, 417 could be retained after removing the duplicates. Ultimately, only 50 articles were retained for final evaluation. The data collected allowed the development of a two-armed model around the physiopathological aspects and potential therapeutic targets, as well as aspects of host immunity, respectively. The model was then compared to data from the HIV literature.

**Conclusion:**

Reported data could contribute to a better understanding of molecular mechanisms of cellular infection by SARS-CoV-2 as well as to a more easy explanation of the action of pharmacological agents used for the treatment, while elucidating intimate mechanisms of the immunity of infected subject.

## 1. Introduction

Coronavirus disease 2019 (COVID-19) [1, 2] is an emerging infectious disease of viral zoonosis type, caused by SARS-CoV-2, a virus belonging to coronaviridae family, identified in Wuhan, China, and responsible for a pandemic declared since December 2019 [3]. Crown-shaped virus initially noted 2019-nCoV and subsequently SARS-CoV-2 [3]. The global spread, aided by international traffic flows, has facilitated disease spread in the world. Today, no continent is spared from this disease. The first cases were reported in Hubei province, China, among seafood vendors hospitalized with rapidly progressing pneumonia [4]. The improvement and rapid development of molecular biology techniques (multiplex PCR, sequencing, etc.) have enabled Chinese researchers to target very quickly, in less than 3 days, nearly 22 respiratory pathogens (18 viruses and 4 bacteria). The negative results of this approach suggest the possibility of a new respiratory pathogen [5]. The molecular characteristics revealed the viral nature of the germ, very close genetically to SARS-CoV-1 and other already known SARS, but the different epidemiological profile of the disease, led to suspect certain phylogenic specificities. The condition was notably marked by a very high and uncontrollable lethality [6–17]. This situation of generalized panic justifies the development of numerous therapeutic and prevention trials using candidate vaccines on a global scale, and more often, through compassionate clinical trials. Political measures, in particular territorial confinement, have been adopted in several countries, with multifaceted consequences, forcing nations to drastic containment measures, to very extensive rapid clinical trials, both therapeutic and vaccine, without any effective solution, which could stop for now its spread. In this documentary review, we sought to describe the state of knowledge on virological data, molecular, and pathophysiological mechanisms of SARS-CoV-2, with a view to better understanding the therapeutic targets, as well as the infected subject's immune response.

## 2. Methodology

**Figure 1:**
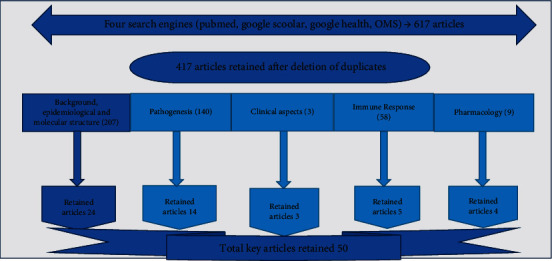
Flow chart of selecting articles.

This literature review is a compilation of several meta-analyses, in accordance with the methodology described in the PRISMA statement on data from the literature on SARS-CoV-2. The articles concerned are those published between March 22 and August 14, 2020, without language restriction [18]. The search engines used for the selection of articles were as follows: PubMed, Google Scholar, Global Health, and WHO reports. The articles of interest were those dealing with virological and molecular data on SARS-CoV-2, the therapeutic aspects of COVID-19, and the immunity of the infected subject. Of the 617 eligible articles, 417 could be retained after removing the duplicates. Only 50 articles meeting the requirements and related to the proposed themes were selected for the final evaluation. The flow of selecting items is shown in the diagram below (Figure 1). The data collected allowed the development of a two-armed model around the physiopathogenic aspects and potential therapeutic targets, as well as aspects of the immunity of the infected subject, respectively. Eligibility of articles was verified by two independent reviewers, with use of a third opinion in case of discrepancy.

## 3. Results and Comments

### 3.1. Generalities on Epidemiology and Molecular Structure of SARS-CoV-2

**Figure 2:**
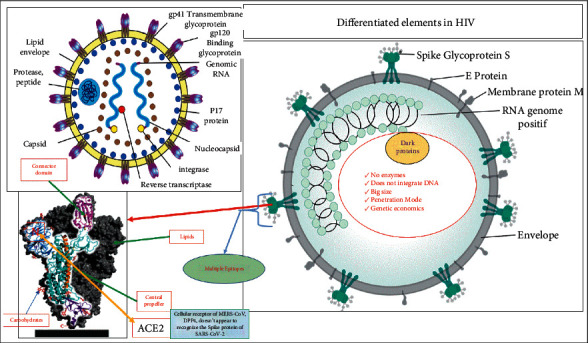
Structure and differentiated elements between SARS-CoV-2 and HIV (Sources: UCL/AUF; https://en.wikipedia.org completed by this study).

The results of this systematic review led to the proposal of two new regimens: one on physiopathogenic aspects and potential therapeutic targets and the other on aspects of host immunity (Figure 2). This new model is compared to data from the HIV literature. Serological curves of IgM/IgG antibodies are compared between subjects with primary infection versus anamnestic or secondary reaction. Figure 2 illustrates the morpho-molecular structure of SARS-CoV-2. Like all coronaviruses, SARS-CoV-2 has the shape of a crown, but it has in its composition two capital elements, namely, the spike (S) protein and its genetic material, which is an ssRNA, that is, single-stranded called RNA positive. [19–21]. In addition to these two elements, the virus contains many proteins playing a role in its constitution detailed in Figure 2: membrane, envelope, nucleocapsid (M, E, N, etc.), and inside there are also obscure proteins.

The comparison of HIV reveals that SARS-CoV-2 has no enzymatic mechanism. Indeed, HIV contains enzymes such as reverse transcriptase (RT) and integrase, enzymes that allow it to reverse transcribe to generate negative RNA, and integrase that will help it to integrate host DNA. It is the machinery that allows the HIV replication cycle [22–25]. The situation is totally different for SARS-CoV-2, which does not contain any enzymes. Apart from its spike, a penetrating element recognized by ACE2 receptors, all the other elements are found in its positive RNA in the form of genes or nucleotides [26–33]. The open reading frames (ORFs) 1a to 1b genes will be translated by the ribosome into replicase, an ARN polymerase responsible for the synthesis of a negative-strand RNA from a positive-strand RNA [34, 35].

SARS-CoV-2 never integrates host cell DNA, but his genomic RNA has a 5-methylated cap and 3-polyadenylate tail that are fully recognized and read by host ribosomes [34, 36]. This gives impression that positive RNA of virus came from the process of transcription of DNA of human host (which would probably be the basis of controversy that SARS-CoV-2 is an artificial virus). Unlike HIV, which, by integrating the host's DNA, produce RNAs similar to RNAs, normally produced by host's DNA to generate proteins necessary for cellular function [37].

The spike protein plays the role of key, which must be recognized by ACE2 receptor as the lock. This protein is in an inactive form (immature state), and to make it active, the presence of metalloproteinases, including furin and transmembrane serine 2 protease (TMPRSS2), acting as cofactors [38–41], is necessary. This spike protein, taking into account the behavior of the virus and its different infectivity from one person to another, would contain different epitopes (antigenic motifs), some of which are very infectious and others not [42].

### 3.2. The Replication Process, Pathophysiology, and Therapeutic Targets

**Figure 3:**
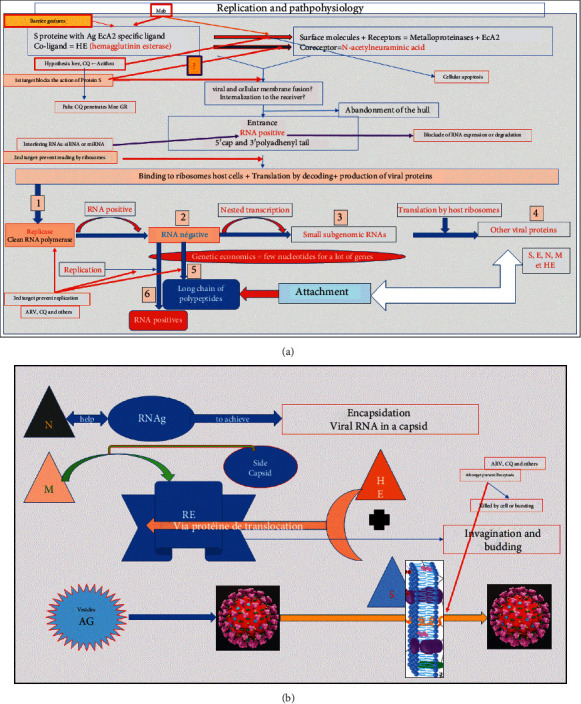
(a) Replication scheme, pathophysiology, and potential therapeutic targets (Source: this study). (b) Diagram (continued) of replication, pathophysiology, and potential therapeutic targets (Source: https://wikipedia.org and this study).

Figures 3(a) and 3(b) indicate the replication process, pathophysiology, and therapeutic targets. In first step and its potential targets, the spike SARS-CoV-2 antigen with its different epitopes is recognized by ACE2 receptors aided by metalloproteinase cofactors (furin or TMPRSS2). At this level, several actions and therapeutic targets are possible: 1a/barrier gestures can prevent the virus from binding and entering the target cells, but antispike monoclonal antibodies (Mab) (a test American in classes) represent the mainly therapeutic target. These Mabs most often target one of the most virulent/infectious epitopes. 1b/Certain hypothetical drugs (hydroxychloroquine (HCQ) + azithromycin (AZT), ivermectin, etc.) having the property of modifying the low PH of endosomes that promote the action of the spike protein. Example of CQ in its form HCQ (adds an–OH group, making it less toxic and tolerable) increases the PH of endosomes and would prevent effective action of spike but also interferes with certain enzymes. 1c/Apoptotic mechanisms of infected cell are also described to prevent the multiplication and contamination of other cells.

**Figure 4:**
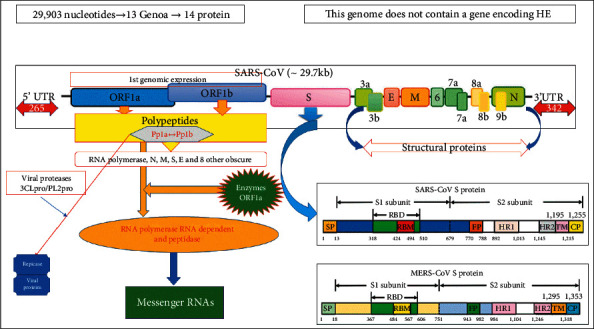
Description of the expressions of reading frames Orf1 to Orf2, protein S, and Orf3a to 9b (Source: https://mdpi.com supplemented by this study).

In second step and its potential targets, the fusion between viral and cellular membranes, most often at the endosome level, will allow entry of positive RNA genetic material in the cytosol where ribosomes (1st organism used by the virus) recognize its 5′methylated cap and its 3′-polyadenylate tail viral RNA as if it was result of a specific transcription of DNA from the host. Ribosomes will have the 1st ORF (Figure 4) on reading and translation and will produce the replicase (RNA polymerase), an essential element of sequence of events. At this 2nd level, the potential therapeutic targets: 2a/viral positive RNA, it is possible to use interfering RNAs, namely, microRNA (mid-RNA) or RNA silencing (Si-RNA). These are small RNAs that complete part of the viral RNA to prevent translation or induce translation. Since SARS-CoV-2 closely resembles other viruses in its family like SARS-CoV-1 at around 80%, Si RNAs would be much easier to design.

In third step and its potential targets, the production of the replicase (RNA polymerase) will act on the positive RNA to generate the negative RNA and the latter by a nested transcription mechanism will produce small genomic RNAs, which will be translated by the ribosomes for the production of other viral proteins (M, S, E, N, etc.). Negative RNA will also produce a long chain of polypeptides and many positive RNAs. At this level, the potential therapeutic targets are as follows: 3a/ARVs such as remdesivir and others which can block either the action of the replicase or other negative RNAs and prevent it from producing genomic RNAs and polypeptides. 3b/Mention will also be made of certain medicaments such as chloroquine, which have shown in vitro an inhibitory activity on the replication of numerous viruses and even by inhibiting the glycosylation of the proteins of numerous enveloped viruses.

In fourth step and its potential targets, assembly of small virions is shown in Figure 3(b). The N protein aided by the genomic RNA will carry out the encapsidation of the viral RNA in a capsid and the M protein enters the ER and goes toward the lateral capsid and the H and E proteins plus the spike S protein enter the ER via a translational protein. All will carry out an invagination and a budding of the small virions, which will finish their maturation at the level of the Golgian vesicles to leave by exocytosis. In this last step, the potential targets come down to blocking the exocytosis of small virions, which will cause the virions suffocation in the endosomes before their probable release. Drugs must be designed to destroy new virions when they emerge.

**Figure 5:**
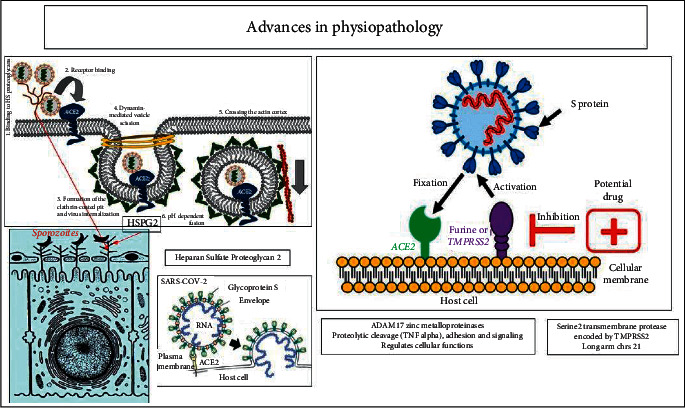
Comparison of the actions of SARS-CoV-2 and Pf sporozoites on HSPG2 (Source https://wikipedia.org and afc.asso.fr).

Progress in the pathophysiology and potential anticipatory targets are shown in Figure 5. We observe a similarity between the action of the S spike of the SARS-CoV-2 virus and the sporozoites of Plasmodium falciparum. Indeed, the SARS-CoV-2 spike protein binds directly the heparan sulfate proteoglycan (HSPG), which facilitates the attachment of viral particles to the cell surface to promote cell entry through the ACE2 receptor. On the hepatocytes, we find the same mechanism with Pf sporozoites. These two actions are qualified as “permissive role” granted by the HSPGs before the binding of the spike antigens S on ACE2 and circumsporozoite proteins (CSPs) of Pf before on hepatocyte receptors. Anti-HSPG antibody can help to preventSARS-CoV-2 infection. In the same sense, antimetalloproteins (antifurin or anti-TMPRSS2) can be produced to prevent the maturation and, subsequently, the activation of the spike protein.

### 3.3. Immune Response of the Subject Infected with SARS-CoV-2

**Figure 6:**
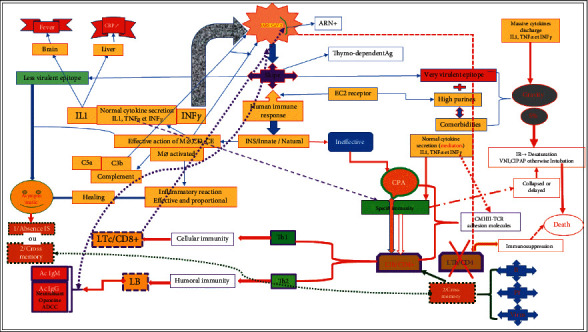
Immune response of the infected host and the different situations (Source: this study).

The immune response to SARS-CoV-2 was initially the subject of several controversies that are increasingly being dispelled following clarification from several studies. In Figure 6, we try to elucidate the response of the subject infected with SARS-CoV-2 with several facets and the possible potential therapeutic targets. It should also be recognized on board that the spike antigen would present several epitopes (determinants or even antigenic patterns) that are different from each other. Among its many epitopes, there are some which are less virulent, some avirulent, and finally others very virulent. The variability in the nature of its epitopes (avirulent, moderately virulent, and very virulent) could also be correlated with the severity and the speed of the infection without taking into account the capacity of the variable immune response and the genetic susceptibility of the infected individuals.

First case of species if the individual is in contact with a spike having all avirulent or less virulent epitopes, the action of nonspecific immunity cells (INS) by macrophages, dendritic cells through their action, and phagocytosis would be effective in neutralizing the virus, certainly with the help of certain cytokines such as gamma interferons secreted by its macrophages. Consequence of this action would be characterized by the absence of the immunity memory except for certain people who having been in contact with other attackers having the same types of epitopes. These people could develop a specific immunity with memory that we can qualify as “cross.” This cross-immunity was reported in malaria-endemic belts based on the fact that SARS-CoV-2 and plasmodium falciparum share common immunodominant epitopes. Indeed, to invade the hepatocytes, the sporozoites of plasmodium falciparum present two antigens, the circumsporozoite protein (CSP) and the thrombospondin-related anonymous protein (TRAP), which interferes with prominent glycosaminoglycans (GAGs) in hepatic sinusoids. The protein nature of these TRAPs presents similarities with the SARS-CoV-2 epitopes (N and open reading frame (ORF)1ab) explaining the low incidence of COVID-19 case severity and mortality in African countries and tropical regions [43].

2nd scenario: if the spike antigen has very virulent epitopes and the host cell is genetically loaded with an excessive amount of metalloproteins (furin or TMPRSS2) and, added to this, the presence of comorbidity, the action of activated macrophages with a significant cytokine discharge (cytokine storm?) will explain the severity of COVID-19 in 5% of cases requiring noninvasive ventilation (NIV = CIPAP) at the start and otherwise intubation, which most often lead at death in over 90% of cases. There is also a constant delay or collapse of the specific immune system (SI), which does not respond due to multiorgan failure. It is increasingly recognized that these epitopes have the ability to weaken the action of the specific immune response by neutralizing T-helper (CD4 +) lymphocytes, which will result in immunosuppression. The latter is believed to be correlated with important biological signs related to the severity of the disease, which, moreover, are considered to be important biological markers of SARS-CoV-2 infection. Mention may be made on lymphopenia less than 1,500 lymphocytes/ml, lymphocytopenia (following an apoptotic action of lymphocytes), thrombocytopenia less than 150,000 platelets/ml similar to that observed in the case of malaria, and leukopenia less than 1,500 leukocytes/ml [44, 45]. Other abnormalities are also described as impact on other organs including kidneys and liver [46, 47].

Third scenario: spike antigen with thymo-dependent epitopes eliciting an initially nonspecific immune response with an action of activated macrophages secreting cytokines IL-1, IL-6, TNF*α*, and INF*γ*. These cytokines are secreted to complement the inflammatory reaction with complement. They act (IL-1, INF*γ*, and TNF*α*) on the brain and the liver. In the brain, on the hypothalamic-pituitary axis, IL-1 and TNF*α* act on the thermoregulatory center to generate fever.

In the liver, they cause increased secretion of the CRP protein, which is a sign of inflammation. IL-1 cytokines also activate the complement system alternately to produce C3b, which opsonizes the virus by facilitating phagocytosis by macrophages. Then, C5a by its chemotactic action would lead to a massive influx of phagocytic cells (polynuclear neutrophils, basophils, monocytes, etc.) to amplify the inflammatory reaction, which, if effective, can lead to a cure. But if this response proves ineffective, macrophages, dendritic cells, and others can express the major histocompatibility class II complex (MHCII) to become antigen-presenting cells (APC) able to present the SARS-CoV-2 antigen to the TCR receptors of Th/CD4 + lymphocytes. Normally, some SARS-CoV-2 viruses can act directly against Th/CD4 + lymphocytes and weaken them and this will be the basis of an immunosuppression that can be fatal for the host. This breakdown in specific immunity is seen in patients with increased desaturation and in invasive ventilation (intubation) can last from 3 weeks to 1 month without success and results in death.

Collaboration between APCs and LyTh/CD4 +  is mediated by cytokines taking the Th1 versus Th2 pathway. The activated LyTh/CD4 +  will proliferate and differentiate into two types of lymphocytes population (effectors and memories). The memories in small numbers are captured by the image of the SARS-CoV-2 antigen and withdraw for an anamnestic response and the effectors, which have a cytotoxic effect on viruses, will act by the Th1/cellular pathway on Ly Tc/CD8 +  (memories and effectors). Then, we have the Th2/humoral pathway where the LTh acts under the action of cytokines on the B lymphocytes, which will multiply to give the B memories in small number and the effectors, in large number which will differentiate into plasma cells to produce first the antibody IgM and then the neutralizing IgG. The latter are opsonins capable of blocking the SARS-CoV-2 Ag and facilitating phagocytosis by macrophages thanks to their RFc receptor and this through an ADCC power (antibody cell cytotoxicity). The cellular route appears to be more efficient than the humoral route because some Ab have not shown great affinity and dilute rapidly in serum to disappear after 3 months to 1 year. It is assumed that certain aggressors (bacteria, viruses, and parasites) present more in tropical areas would have certain epitopes identical to those of SARS-CoV-2 and these people would have developed a specific immunity with LyTh/CD4 + memories, LyTc/CD8 + memories, LB memories, and memory IgG Ab without having been in contact with SARS-CoV-2. Figure 6 describes the different situations of the immune response of the infected host.

**Figure 7:**
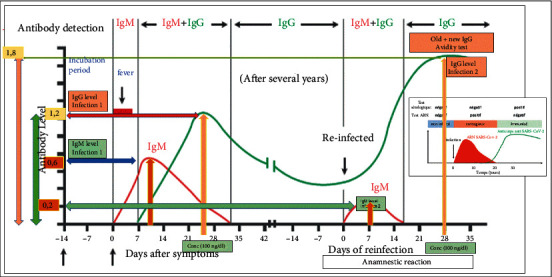
Serological curves of the evolution of IgM Ab/IgG primary invasion versus secondary or anamnestic reaction (Source: CliniSciences supplemented by this study).

Figure 7 describes on the *x*-axis the evolution of IgM and IgG Ab and on the *y*-axis the quantified rate of production of its Abs.

#### 3.3.1. Situation of Primary Invasion (First Infection) with SARS-CoV-2

After an incubation period of about 7 days, the humoral response as by the production of IgMs, which reach their peak at about 10 days at the rate of production of around 0.6, will start to drop for complete disappearance around the 31st or 32nd day. On the other hand, the IgG Ab starts their production from the 7th day and reaches their peak around the 25-26th day at the rate of 1.2 and begin to decrease beyond the 42nd day around the 63rd day to stabilize at a rate of 0.4, which attests a certain memory between 3 months and 12 months from one individual to another.

#### 3.3.2. Situation of Reinfection (Second Infection with an Anamnestic Reaction) with SARS-CoV-2

The specific immune reaction is more refined, faster with production of IgM antibodies reaching their peak more quickly from the 7th day at a low rate of 0.2, whereas, in the first invasion, it was rather around the 10th day and decrease to disappear completely around the 16th day, so they could persist beyond the 25th day. On the other hand, the IgG Ab well before the 7th day increases in production to reach their peak around the 28th day at the very high rate of 1.8.

#### 3.3.3. Comments

People presenting as positive for SARS-CoV-2 with the rapid test can be confusing and could all be considered as primary invasion, while some may be in a situation of second infection/reinfection due to the multiple interpretations of the Ab IgM results/IgG positive or negative but confusion related to the production rate, the duration, and period of appearance or disappearance of its Abs. Ab quantification and avidity tests can help in this sense, in addition to strain genotyping by automatic sequencing of any sample.

**Figure 8:**
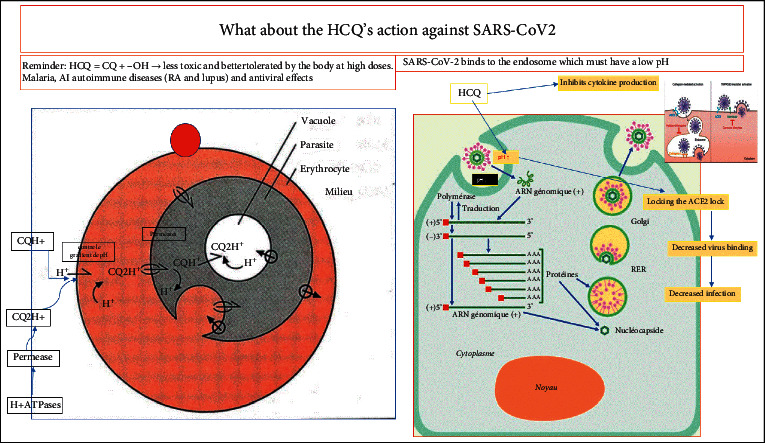
Comparison of HCQ actions in SARS-CoV-2 and Pf trophozoites (Sources: https://wikipedia.org and Futura-Sciences completed in this study).

Figure 8 depicts the comparison of the actions of SARS-CoV-2 and Pf sporozoites on HSPG2.

The two figures show the comparative action of hydroxyl-chloroquine (HCQ) in the plasmodium parasite versus the SARS-CoV-2 virus. Chloroquine being a very toxic product and less tolerated at high doses, and the addition of the hydroxyl (–OH) group makes it less toxic and well tolerated at high doses. CQ in its neutral form is rare about 0.2% and is permeable to the plasma membranes of most cells. CQ or HCQ is presented in the majority of cases in mono or di-protonated form (CQH + or CQH2 +), which makes it impermeable to cell membranes. Therefore, their entry will require the contribution of permeases or translocases (lactose permease and beta-galactoside permease) and the latter also need support by H + ATPases to help them facilitate the entry of mono or protonated HCQ.

1. During malaria infection, HCQ crosses the parasitized GR membrane thanks to the action of permeases (themselves help by H + ATPases), which allows it to penetrate and go to concentrate in the parasitophorous vacuole and block the Hb captured by the trophozoite. This eventually results in the death of the parasite, and the released Hb will be able to return to the bloodstream again and pair with oxygen.
2. SARS-CoV-2 takes advantage of a low pH in the endosomes, which facilitates its infectivity, but the action of HCQ is to increase this pH, which has a negative impact on the evolution of the virus. In addition, in its action, HCQ is described as having an anticytokine effect, and in vitro, this molecular has shown antiviral actions against viral RNAs or their replicases [48–50].

## 4. Conclusion

This review allows us to understand the concept of the epitopes of the spike protein generating antigenic variants of SARS-CoV-2, which turn out to be at the basis of various immunopathological responses in the infected subject. Potential therapeutic targets, some of which have shown their ineffectiveness or paradoxical efficacy, and others are the avenues of solution currently being exploited. Finally, this meta-analysis also shows the benefit of knowing the different variants (epitopes) and of producing multivariate vaccines to control the COVID-19 pandemic.

## Data Availability

The data used to support the findings of this study are available from the corresponding author upon request.
